# Clinical Characteristics, Histopathological Profile, and Postoperative Outcomes of Tumoral Radiculomedullary Compressions

**DOI:** 10.7759/cureus.83333

**Published:** 2025-05-02

**Authors:** Israël Maoneo, Larrey Kasereka Kamabu, Chérubin Tshiunza, Ntalaja Jeff, Kambere Renault, Bienvenu Lebwaze, Raphaël Chirimwami, Antoine Beltchika, Glennie Ntsambi

**Affiliations:** 1 Neurosurgery, University of Kisangani, Kisangani, COD; 2 Neurosurgery, Catholic University of Graben, Butembo, COD; 3 Neurological Surgery, New Deal Sarl Hospital, International Clinic for Advanced Medicine in Kivu (CIMAK), Goma, COD; 4 Neurological Surgery, Makerere University, Kampala, UGA; 5 Neurosurgery, Centre Hospitalier Initiative Plus, Kinshasa, COD; 6 Neurosurgery, Clinique Ngaliema, Kinshasa, COD; 7 Pathological Anatomy, University of Kinshasa, Kinshasa, COD; 8 Neurosurgery, University of Kinshasa, Kinshasa, COD

**Keywords:** non-traumatic paraplegia, non-traumatic radiculo-medullary compressions, non-traumatic spinal cord injuries, spinal tumoral compression, spinal tumors, spinal tumor surgery

## Abstract

Background and objectives

Spinal tumors can cause slow radiculomedullary compression and often lead to severe neurological dysfunction for the patient. The objective of this study was to describe the clinical characteristics, histopathological profile, and postoperative outcomes of tumor-related radiculomedullary compressions.

Methods

This was a prospective cohort study conducted from January 2020 to June 2024 concerning cases of spinal tumors responsible for a radiculomedullary compressive syndrome at the Department of Neurosurgery, University Teaching Hospital of Kinshasa. The variables of interest included sex, age, cause, level of the lesion, the American Spinal Injury Association (ASIA) score, the Spinal Cord Independence Measure (SCIM) score, treatment, postoperative outcome, and complications.

Results

Thirty-four patients were operated on for tumor-related radiculomedullary compression. There were 19 males and 15 females, and the male-to-female ratio was 1.2:1. Patients under 50 years represented 52.9% of cases. The average age was 48.06 ± 18.92 years. Most patients presented with pain at the lesion site (64.7%), incomplete paraplegia (76.6%), and bladder-sphincter dysfunction (41.2%). The average preoperative delay was 124.18 ± 73.34 days. Less than one-third of the patients (29.4%) had comorbidities. Twenty-six patients (76.6%) were classified as ASIA grade B, and 27 patients (79.4%) had a SCIM score below 40. Most lesions were located in the thoracic (55.9%) and lumbar (36.3%) segments. Nineteen tumors (55.9%) were intradural extramedullary, 14 were extradural (41.2%), and one was an intramedullary tumor, an ependymoma (2.9%). There were 20 benign lesions (58.8%) and 14 malignant lesions (41.2%). Benign lesions, mainly meningiomas (47.1%), predominated in female patients (p = 0.002), with no age preference (p = 0.082). Malignant tumors predominated in male patients (p = 0.002) over the age of 50 years (p = 0.017), all of which were metastases (14/14: 100%), mostly from prostate adenocarcinoma (64.2%), followed by breast adenocarcinoma (21.4%). Patients with metastatic compression had 16 times higher risk of worsening or persistent neurological deficits (OR: 16.32, CI: 2.61-100.44, p = 0.003) compared to those with benign tumors (OR: 0.06, CI: 0.01-0.38, p = 0.003).

Conclusion

This study shows that benign tumors are more frequent than malignant tumors as causes of tumor-related radiculomedullary compression operated on in our department. Meningiomas predominate among intradural extramedullary tumors. Metastases are the most frequent malignant tumors, with prostate adenocarcinoma being the most common histopathological type, often associated with poor neurological outcomes.

## Introduction

Spinal tumors include extradural and intradural tumors. These can be further classified into extramedullary and intramedullary tumors. Extradural tumors are the most common spinal tumors, accounting for 50% of cases [1,2]. They may be primary or secondary. Secondary tumors are by far the most frequent, representing 97% of all vertebral tumors [3]. Additionally, 70% of cancer patients develop metastases to the spine [4,5]. Primary extradural tumors are extremely rare. These primarily include hemangiomas and exostoses, with an incidence of 11-14% [3]. They are often asymptomatic and discovered incidentally [2].

Intradural extramedullary tumors are the second most common spinal tumors, making up 40% of cases [2]. They develop from the arachnoid cap cells or nerve roots [6]. Their frequency varies across study series, but typically there are schwannomas (42.3%), followed by meningiomas (28.4%) and neurofibromas (12.3%) [7].

Magnetic resonance imaging (MRI) is the gold standard for diagnosing intradural tumors, determining their size, location, and axial position [2,8,9]. Myelography or computed tomography (CT) may be used to identify calcification within the lesion or when MRI is contraindicated. Spinal angiography is often performed prior to surgical intervention for spinal meningiomas in the thoracolumbar region to identify the artery of Adamkiewicz in relation to the lesion [8,9].

The most frequent cause of neurological dysfunction is radiculomedullary compression. Extradural metastatic bone lesions may be osteolytic, involving the destruction of normal bone, or osteoblastic due to the deposition of new bone. Both types of lesions can cause instability of the vertebral body. Bone instability may lead to the retropulsion of bone fragments into the epidural space after vertebral body collapse. Furthermore, the tumor itself may grow and encroach anteriorly into the thecal sac, thereby compressing the spinal cord, nerve roots, and epidural venous plexus. The development of intradural extramedullary and intramedullary lesions can also compress the radiculomedullary neurovascular structures [10].

Tumor-related radiculomedullary compression is a critical public health issue, separate from the underlying tumor disease itself. Patients become dependent and may develop complications that can lead to death. The current therapeutic protocol for spinal tumors recommends surgical resection aimed at completely removing the tumor and decompressing the spinal cord [11]. Adjuvant radiotherapy, in addition to surgery, has been reported in the management of recurrent and atypical/anaplastic tumors [12].

The success of surgical excision has improved due to advances in neuroimaging, microsurgical techniques, the use of ultrasonic aspirators, and intraoperative electrophysiological monitoring [2]. However, such advanced surgical resources are not often available in resource-limited settings such as the Democratic Republic of the Congo. In general, spinal tumors are under-documented in most African countries. This may be due to limited diagnostic capabilities, often leading to delayed diagnoses, therapeutic challenges, and a shortage of personnel necessary for the diagnosis, surgical management, and documentation of these tumors.

The objective of this study was to characterize the clinical and histopathological profiles of spinal tumors, to evaluate postoperative neurological (American Spinal Injury Association (ASIA) score) and functional outcomes (Spinal Cord Independence Measure (SCIM) score) under the working conditions of the Department of Neurosurgery, University Teaching Hospital of Kinshasa.

## Materials and methods

Study design

This was a prospective cohort study conducted at the Teaching Hospital of Kinshasa from January 1, 2020, to June 30, 2024. During this period, 38 patients with spinal tumors were hospitalized and underwent surgery. Only the records of 34 patients were included, as they met the selection criteria.

Data collection technique

We used a prospective documentary data collection method utilizing hospitalization and operating room registers, as well as a data collection sheet.

Inclusion Criteria

All patients admitted and operated on at the Spinal Cord Injury Unit (SCIU) with a tumor-related radiculomedullary compression syndrome between January 2020 and June 2024 were included in this study. We included patients with spinal cord compressions (C1-L2) and those with root compressions (L3-S2).

Exclusion Criteria

Patients admitted and operated on at SCIU for tumor-related radiculomedullary compression between January 1, 2020, and June 30, 2024, whose data were inaccessible or who had previously undergone spinal surgery for another traumatic or non-traumatic cause were excluded from the study.

In addition to socio-demographic and clinical variables (sex, age, date of admission, surgery date, discharge date, profession, patient complaints, medical history, vital signs, cause of radiculomedullary compression, vertebral level of the lesion, neurological level, treatment, complications, and post-discharge destination), we determined the histopathological nature of the spinal tumors and the ASIA and SCIM scores of the patients before surgical treatment. Patients were then followed postoperatively with monthly assessments of their ASIA and SCIM scores until discharge.

Therapeutic aspects

Surgical Procedures

We typically performed a two- or three-level laminectomy depending on tumor extent. For extradural tumors, we excised the tumor or removed bone metastases, collected biopsy specimens, and closed the muscles, fascia, subcutaneous tissue, and skin, with or without suction drainage. For intradural lesions, we performed a 3-4 cm longitudinal durotomy depending on tumor size, opening the outer dural layer with micro-instruments, extending proximally and distally beyond the tumor.

Tumor dissection involved carefully separating the inner dural layer and nerve roots from the tumor using Saito’s technique [13] and micro-instruments. After complete tumor resection and dural sac decompression, hemostasis was achieved with Surgicel, followed by watertight closure of the outer dural layer using 3/0 Vicryl without biological glue. A Porto-Vac drain was left in place, and soft tissues were sutured in layers. In cases of spinal instability, transpedicular fixation was performed.

Medical Treatment

All patients received analgesics, anti-inflammatory drugs, gastric antisecretory agents, postoperative corticosteroids, neurotropic drugs, anticoagulants, and antibiotic coverage. For malignant tumors, anticancer drugs were added per oncological protocols.

Radiotherapy

For some cases of vertebral metastases, radiotherapy sessions were conducted before and after surgery at the Nganda Hospital Radiotherapy Center in Kinshasa.

Physiotherapy

All patients were followed by the physical medicine team for various rehabilitation procedures (physiotherapy, occupational therapy, etc.) postoperatively until discharge. Some patients continued rehabilitation in specialized centers after discharge.

Microscopy

Intraoperative samples were placed in labeled jars containing 10% formalin and sent to the pathology lab at the University Teaching Hospital of Kinshasa. In the lab, samples were examined macroscopically (external and internal aspects), placed into cassettes with matching numbers, and cut into shreds of 3 to 5 micrometers. The shreds obtained were spread on blades previously coated with albumen and hydrated. The blades were stained first with hematoxylin and then with eosin. After coloring, the blades were read first at objective 10, then 40, and finally 100.

Tumor Grades

After microscopic analysis, tumors were categorized into histopathological grades. The first grade includes tumor cells and tissues that resemble healthy cells or tissues. Tumors are well-differentiated and considered low-grade tumors. The second grade consists of tumor cells and tissues somewhat abnormal and are considered moderately differentiated; classified as intermediate-grade tumors. In the third grade, tumor cells and tissues are very abnormal. Tumors are poorly differentiated and considered high-grade tumors. The fourth grade includes tumor cells and tissues that are highly abnormal. Tumors are undifferentiated and considered the highest-grade tumors [14].

Statistical analyses

Data were recorded and analyzed using SPSS version 26 (IBM Corp., Armonk, NY) and STATA version 17 (StataCorp LLC, College Station, TX) software. A descriptive analysis was performed for the main variables included in the study. Possible associations between the various considered variables were determined using Pearson’s chi-square test or Fisher’s exact test. Odds ratios and logistic regression were used to estimate improvement in ASIA and SCIM scores at hospital discharge. A result was considered statistically significant when the p-value was less than 0.05.

Ethical considerations

This study was approved by the Ethics Committee of the School of Public Health (ESP/CE) at the University of Kinshasa under reference number ESP/CE/043/2023.

## Results

Socio-demographic, clinical, and paraclinical characteristics of patients at admission

Thirty-four patients were included in this study, of whom 19 (55.9%) were male and 15 (44.1%) were female, giving a sex ratio of 1.2:1. Patients under 50 years of age represented 52.9% of cases. The mean age was 48.06 ± 18.92 years. The majority of patients presented with pain (64.7%) related to the location of the lesion, incomplete paraplegia as a neurological deficit (76.6%), and bladder-sphincter disorders (41.2%).

The average preoperative delay was 124.18 ± 73.34 days, approximately four months on average. Less than one-third of patients (29.4%) had comorbidities. Twenty-six patients (76.6%) had an ASIA score of B, and 27 patients (79.4%) had a SCIM score below 40%.

Most lesions were located in the thoracic (55.9%) and lumbar (36.3%) segments. Based on MRI findings, 19 tumors (55.9%) were intradural and extramedullary, 14 (41.2%) were extradural, and one tumor (2.9%) was intramedullary (Table 1).

**Table 1:** Socio-demographic, clinical, and paraclinical characteristics of patients before surgery. SD: standard deviation; ASIA: American Spinal Injury Association; SCIM: Spinal Cord Independence Measure.

Variables	Size (n = 34)	Percentage
Age (years)		
<50	18	52.9
≥50	16	46.1
Mean ± SD (years)	48.06 ± 18.92	
Gender		
Male	19	55.9
Female	15	44.1
Sex ratio	1.2	
Pain	22	64.7
Motor deficit		
Complete paraplegia	1	2.9
Incomplete paraplegia	26	76.6
Paraparesis	7	20.4
Bladder and sphincter disorders	14	41.2
Time between onset of symptoms and surgery (months)		
≤1	5	14.7
>1	29	85.3
Mean preoperative delay ± SD (months)	124.18 ± 73.34	
Comorbidities		
Absent	24	70.6
Present	10	29.4
ASIA score at admission		
A	1	2.9
B	26	76.6
C	7	20.4
SCIM score at admission		
≤40	27	79.4
>40	7	20.6
Mean SCIM ± SD	38.97 ± 14.71	
Spinal level of the lesion		
Cervical	2	5.9
Thoracic	19	55.9
Thoraco-lumbar (T11-L2)	1	2.9
Lumbar	12	36.3
Imaging (MRI)		
Extra-dural	14	41.2
Intra-dural and extra-medullary	19	55.9
Intra-medullary	1	2.9

Therapeutic characteristics and outcomes of treated patients

**Figure 1:**
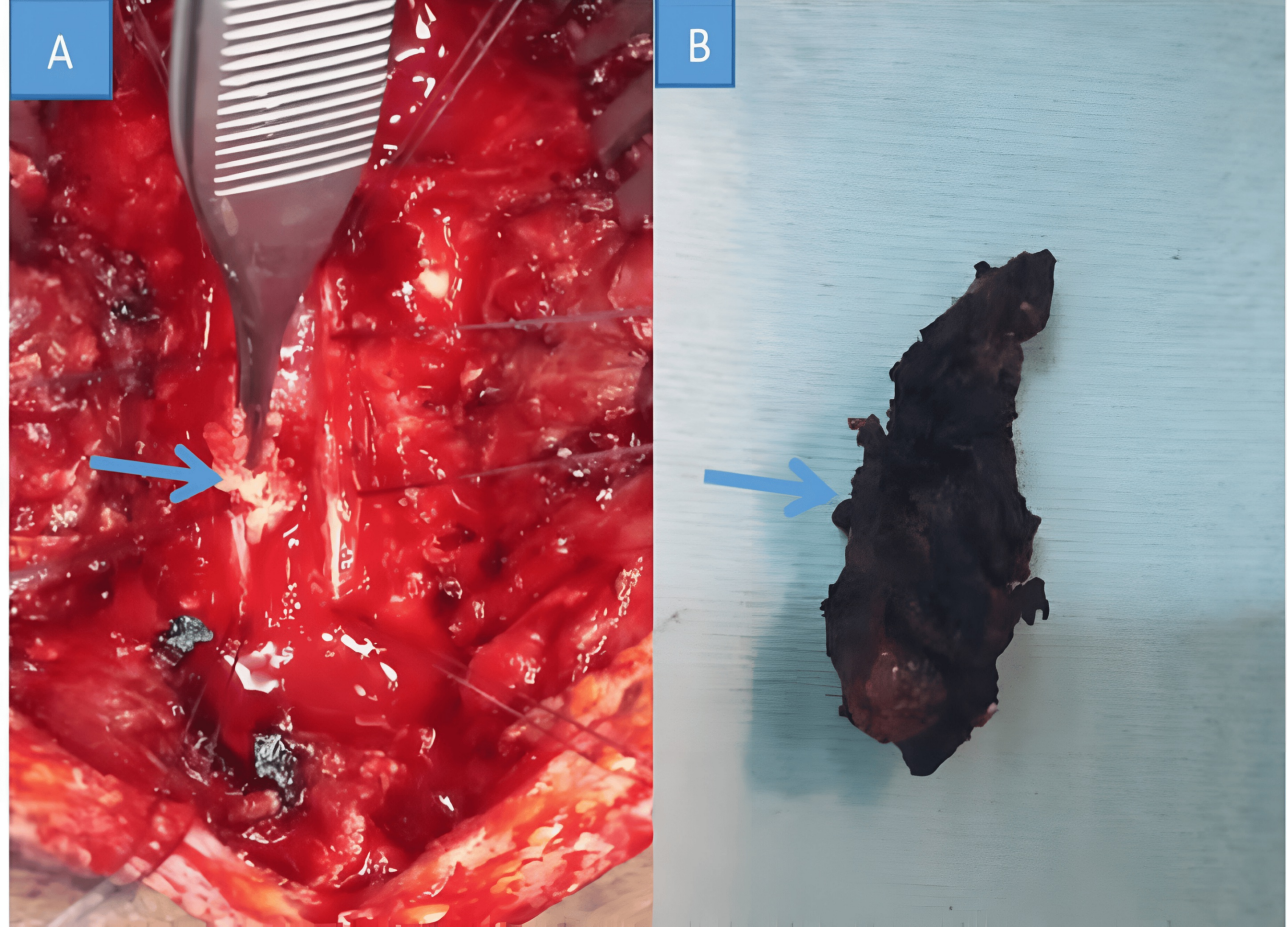
(A) Tumor excision. (B) Resected mass.

The most frequently performed surgical procedure was laminectomy with tumor mass excision (55.9%) (Figure 1). We conducted one laminectomy with tumor excision and fixation (2.9%). A total of 58.8% were gross total excisions. We also performed subtotal resection in 14 (41.2%) patients: laminectomy with biopsy sample in eight patients (23.5%), and laminectomy with fixation in six patients (17.6%). All 34 patients (100%) underwent postoperative rehabilitation sessions. Only eight patients (23.5%) received radiotherapy.

Most patients (55.9%) stayed in the hospital for two weeks after surgery. Nine patients (26.4%) developed complications, including five cases (49.2%) of pressure ulcers, two cases (5.9%) of surgical site infection, and two cases (5.9%) of death. Both patients had died of respiratory distress following the pulmonary swarming of metastasis.

Twenty-three patients (67.6%) improved their admission ASIA score: 13 patients (38.2%) by two grades, seven patients (20.6%) by three grades, and three patients (8.8%) by one grade. Eleven patients (32.4%) retained their initial ASIA score or experienced deterioration.

Twenty-three patients (67.6%) showed favorable progress in relation to their admission SCIM score: 18 out of 27 (66.6%) among those with an initial SCIM score ≤40/100 at admission, and five out of seven (71.4%) in the group with a SCIM score >40/100 (Table 2).

**Table 2:** Therapeutic characteristics and outcomes of treated patients. SD: standard deviation; ASIA: American Spinal Injury Association; SCIM: Spinal Cord Independence Measure.

Variables	Size (n = 34)	Percentage
Surgical treatment		
Laminectomy + mass excision	19	55.9
Laminectomy + biopsy sampling	8	23.5
Laminectomy + spinal fixation	6	17.6
Laminectomy + excision + arthrodesis	1	2.9
Physiotherapy	34	100
Radiotherapy	8	23.5
Complications	9	26.4
None	25	73.5
Pressure ulcers	5	14.7
Surgical site infections	2	5.9
Deaths	2	5.9
Hospital stay (in weeks)		
≤2	19	55.9
3-4	5	14.7
>5	10	29.4
Average hospital stay (±SD)	26.32 ± 17.62	
Outcomes		
ASIA	34	100
B	11	32.4
C	3	8.8
D	9	26.4
E	11	32.4
Improved ASIA score	23	67.6
Grade 1	3	8.8
Grade 2	13	38.2
Grade 3	7	20.6
A	1	2.9
Grade 2	1	2.9
B	17	53
Grade 1	3	8.8
Grade 2	8	23.5
Grade 3	6	17.6
C	5	14.7
Grade 1	0	0
Grade 2	5	14.7
Stable or worsened ASIA score	11	32.4
Improved SCIM score	23	67.6
≤40 (SCIM at admission)	18	52.9
>40 (SCIM at admission)	5	14.7
Average SCIM at discharge (±SD)	71.35 ± 29.99	
Stable or worsened SCIM score	11	32.4

Histopathological data

Types of Tumors by Age and Sex

**Figure 2:**
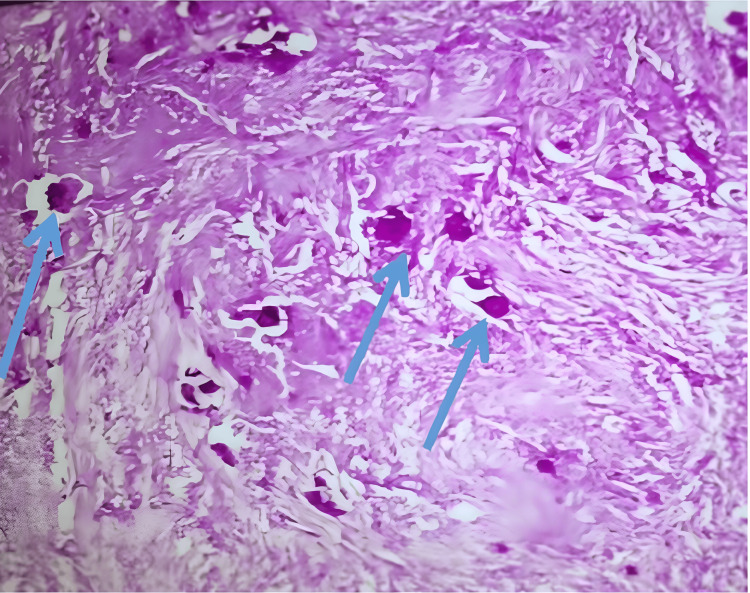
Hematoxylin and eosin-stained image (40x objective) showing a calcified meningioma with psammoma bodies indicated by blue arrows.

**Figure 3:**
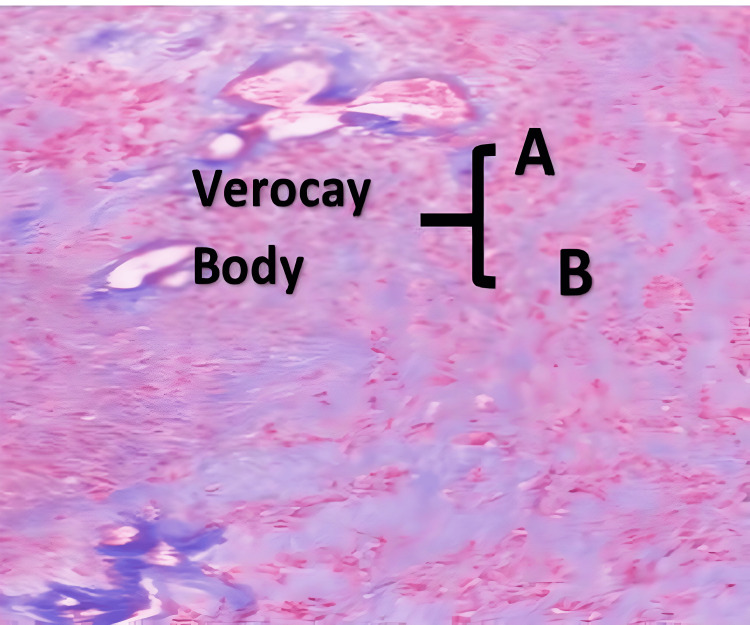
Hematoxylin and eosin-stained image (40x objective) showing a fusocellular proliferation area (Antoni A) and a hypocellular area with myxoid changes (Antoni B). These two areas together form Verocay bodies.

Among the tumor-related causes of radiculomedullary compression, there were 20 benign tumors (58.8%) and 14 malignant tumors (41.2%). Benign lesions predominated in female patients (p = 0.002) under the age of 50 (p = 0.017). The most common benign lesions were meningiomas (47.1%) (Figure 2), while schwannomas accounted for 5.9% (Figure 3), more frequently observed in female patients (p = 0.015), with no specific age preference (p = 0.082). We also diagnosed an extradural fibrolipoma in a 10-year-old female (2.9%).

Malignant tumors predominated in male patients (p = 0.002) over the age of 50 (p = 0.017). All malignant tumors were metastases (14/14, 100%). Prostate adenocarcinoma metastases were the most common (9/14), followed by breast adenocarcinoma (3/14), lung cancer (1/14), and testicular cancer (seminoma) (1/14) (Table 3).

**Table 3:** Types of tumors. * The chi-square test is used to calculate the p-values.

Types of tumors	Sex				Age (years)			
	Male	Female	Total, n (%)	p	˂50	>50	Total, n (%)	P-value^*^
Benign tumors	5	15	20 (58.8)	0.002	14	6	20 (58.8)	0.017
Extradural	0	1	1 (2.9)	0.339	1	0	1 (2.9)	0.339
Fibrolipoma	0	1	1 (2.9)	0.339	1	0	1 (2.9)	0.339
Intra-dural extra-medullary	5	13	18 (52.9)	0.013	15	3	18 (52.9)	0.071
Meningiomas	4	12	16 (47.1)	0.015	14	2	16 (47.1)	0.082
Schwannomas	1	1	2 (5.9)	0.932	1	1	2 (5.9)	0.932
Intra-dural extra-medullary	0	1	1 (2.9)	0.339	1	0	1 (2.9)	0.339
Ependymoma	0	1	1 (2.9)	0.339	1	0	1 (2.9)	0.339
Malignant	11	3	14 (41.2)	0.002	4	10	14 (41.2)	0.017
Primary tumors	0	0	0	0	0	0	0	0
Secondary tumors (metastases)	11	3	14 (41.2)	0.002	4	10	14 (41.2)	0.017
Extra-dural (vertebral)	11	3	14 (41.2)	0.002	4	10	14 (41.2)	0.017
Prostate adenocarcinoma metastasis	9	-	-	-	0	9	9 (26.5)	˂0.001
Breast adenocarcinoma metastasis	0	3	3 (8.8)	0.087	3	0	3 (8.8)	0.087
Seminoma metastasis	1	-	-	-	1	0	1 (2.9)	0.339
Lung cancer metastasis	1	0	1 (2.9)	0.282	0	1	1 (2.9)	0.339

Of 16 meningiomas, 15 were of WHO grade I and one was of grade II. The two cases of schwannoma and one case of fibrolipoma were of grade I. Ependymoma was of grade III. Of nine prostate carcinomas, three were of grade III and six were of WHO grade IV. Two breast carcinoma metastases were of grade IV, and one case was of grade IV. Seminoma metastasis was of grade IV, and lung metastasis was of grade III.

Spinal Localizations

**Figure 4:**
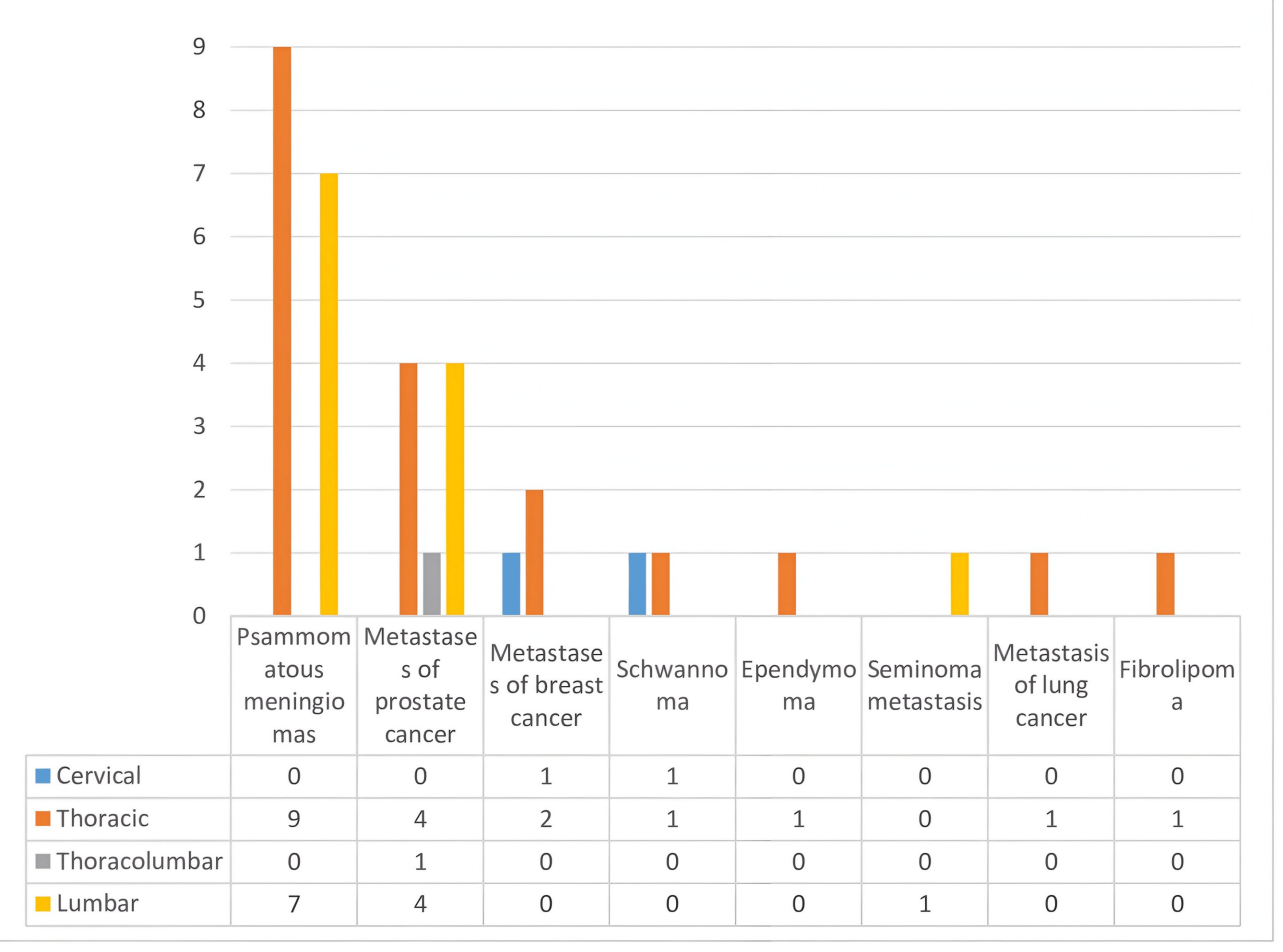
Localization of spinal tumors.

**Figure 5:**
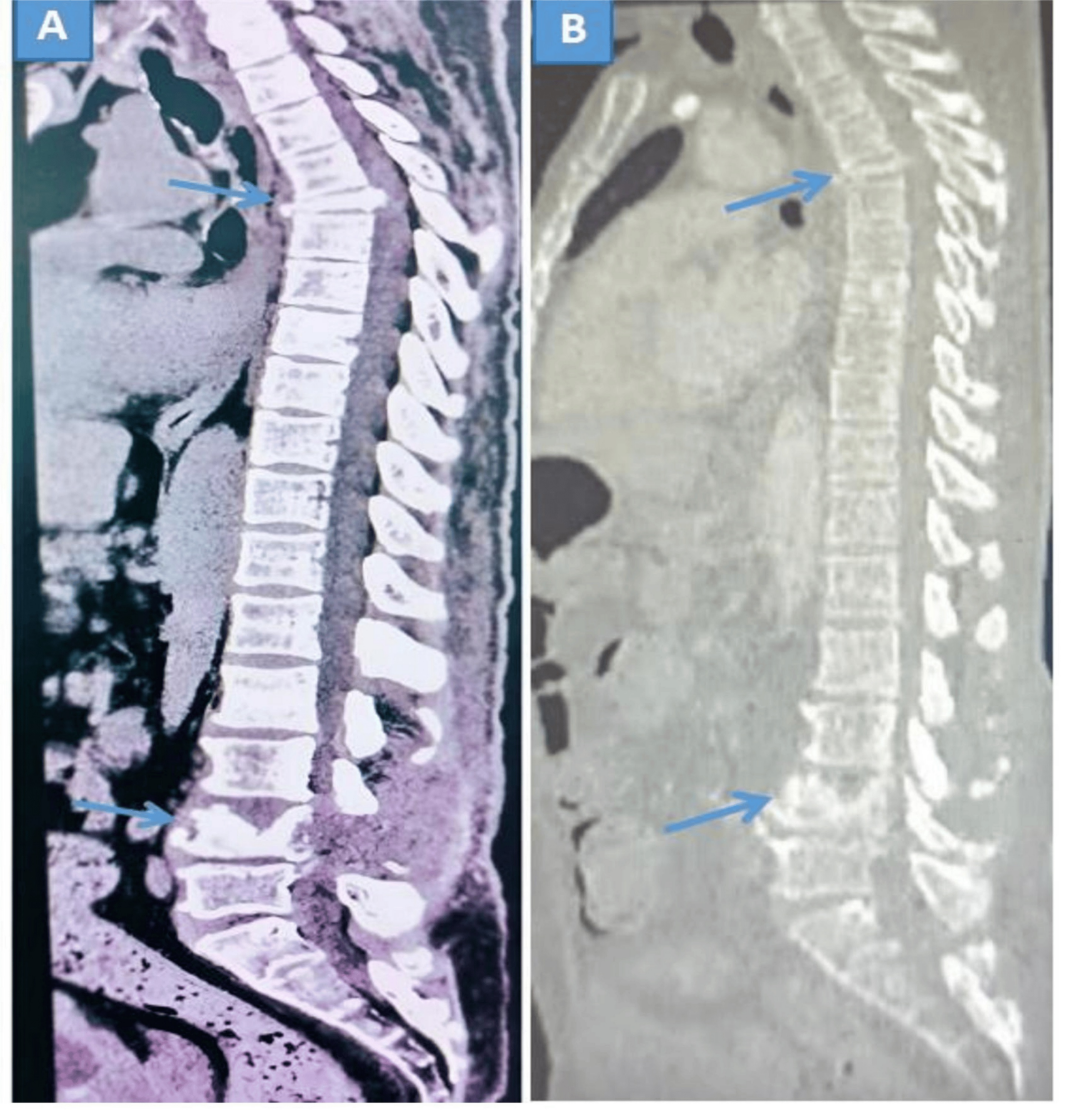
(A) Soft tissue and (B) bone windows of the spinal sagittal section CT scan image showing the thoracic (T5) and lumbar (L4) localization of prostate carcinoma metastases.

Nine meningiomas were localized in the thoracic region (56.3%) and seven in the lumbar region (43.7%). Schwannomas were equally distributed between the cervical (50%) and thoracic (50%) regions. Prostate adenocarcinoma metastases invaded both the thoracic (44.4%) and lumbar (44.4%) segments equally. Breast cancer metastases were mostly found in the thoracic region (66.7%), followed by the lumbar segment (33.3%) (Figure 4). We noted a case of prostate cancer metastasis located in two different areas of the thoracic and lumbar spine (Figure 5).

Postoperative Complications

In most cases, there was no significant difference between the histological types of tumors concerning postoperative complications (Table 4).

**Table 4:** Postoperative complications by histological tumor type. The chi-square test is used to calculate the p-values.

Tumor types	Complications				Total	p-value
	No.	Surgical site infections	Pressure sores	Deaths		
Benign tumors	15	1	2	2	20	0.530
Meningiomas	13	1	1	1	16	0.631
Schwannomas	2	0	0	0	2	0.858
Fibrolipoma	0	1	0	0	1	˂0,001
Ependymoma	0	0	1	0	1	0.113
Malignant tumors	10	0	2	1	14	0.530
Prostate adenocarcinoma metastasis	6	0	3	0	9	0.221
Breast adenocarcinoma metastasis	2	0	0	1	3	0.178
Seminoma	1	0	0	0	0	0.946
Lung cancer metastasis	1	0	0	0	0	0.946

Evolution of Neurological Function

General evolution of the ASIA score: In univariate analysis, patients with metastases were 16 times more likely not to improve their neurological function (OR: 16.32, CI: 2.61-100.44, p = 0.003) compared to those suffering from benign tumors (OR: 0.06, CI: 0.01-0.38, p = 0.003). Patients with prostate adenocarcinoma metastases had an eight times increased risk of maintaining their initial ASIA score or deteriorating (OR: 8.00, CI: 1.46-43.67, p = 0.016). Patients with meningiomas had a 95% chance of improving neurologically (OR: 0.05, CI: 0.01-0.49, p = 0.010) (Table 5). After adjustment in multivariate analysis, benign tumors in general and meningiomas in particular remained good prognostic factors (OR: 0.07, CI: 0.01-0.74, p = 0.027 and OR: 0.83, CI: 0.01-0.93, p = 0.044, respectively), and metastases in general were a poor prognostic factor (OR: 13.50, CI: 1.34-135.89, p = 0.027) (Table 6).

**Table 5:** Evolution of the ASIA score. ASIA- refers to patients whose neurological status remained stationary or worsened. ASIA+ refers to patients who showed improvement. Odds ratio (OR), confidence interval (CI), and p-values indicate the strength and significance of associations in univariate analysis. The chi-square test was used to calculate the p-values. ASIA: American Spinal Injury Association.

Tumor types	ASIA- (stationary or worsened)/ASIA+ (improved)	Univariate analysis		
		OR	CI	p-value
Benign tumors	ASIA-/ASIA+	0.06	0.01-0.38	0.003
Meningiomas	ASIA-/ASIA+	0.05	0.01-0,49	0.010
Schwannomas	ASIA-/ASIA+	1.52	1.18-1.95	0.313
Fibrolipoma	ASIA-/ASIA+	2.50	0.62-9.99	0.186
Ependymoma	ASIA-/ASIA+	3.30	1.96-5.53	0.142
Malignant tumors	ASIA-/ASIA+	16.32	2.61-100.44	0.003
Prostate adenocarcinoma metastasis	ASIA-/ASIA+	8.000	1.46-43.67	0.016
Breast adenocarcinoma metastasis	ASIA-/ASIA+	4.88	0.39-60.92	0.183
Seminoma metastasis	ASIA-/ASIA+	3.30	1.96-5.53	0.142
Lung cancer metastasis	ASIA-/ASIA+	1.50	1.17-1.90	0.483

**Table 6:** Multivariate analysis. * ASIA-: No improvement or deterioration. ASIA+: Improved. The chi-square test was used to calculate the p-values. The covariates used for adjustment were age, sex, preoperative delay, and tumor location. The confounder comorbidities or preoperative ASIA/SCIM scores were included. ASIA: American Spinal Injury Association; SCIM: Spinal Cord Injury Unit.

Types of tumors	*ASIA-/ASIA+	Univariate analysis			Multivariate analysis		
		OR	CI	p-value	OR	CI	p-value
Benign tumors		0.02	0.01-0.27	0.002	0.07	0.01-0.74	0.027
Meningiomas		0.05	0.06-0.49	0.010	0.83	0.01-0.93	0.044
Malignant tumors		36.00	3.67-352.64	0.002	13.50	1.34-135.98	0.027
Prostate adenocarcinoma metastasis		8.000	1.46-43.67	0.016	2.57	0.37-16.88	0.347

Evolution of ASIA grades from admission to discharge: In general, patients with benign tumors showed significant neurological improvement, gaining two to three grades compared to their initial state, with only one patient whose neurological status remained unchanged (p < 0.001). For instance, in meningioma cases, patients gained two to three ASIA grades compared to their initial status. Conversely, patients who developed metastases experienced a significant unfavorable evolution, maintaining their initial neurological state with only small improvements in higher grades. No patient gained three grades. In the cases of prostate adenocarcinoma metastasis, seven patients (77.8%) maintained their initial ASIA grade, while two patients gained two ASIA grades higher than their initial neurological status (Table 7).

**Table 7:** Evolution of ASIA grades from admission to discharge. Grade improvements refer to the number of ASIA grade levels the patient improved between admission and discharge. "0" means no improvement, "1" means improvement by one grade, and so on. The chi-square test was used to calculate the p-values. ASIA: American Spinal Injury Association

Tumor types	Grade improvements				Total	p-value
	0	1	2	3		
Benign tumors	2	0	11	7	20	˂0.001
Meningiomas	1	0	9	6	16	0.001
Schwannomas	0	0	2	0	2	0.330
Fibrolipoma	0	0	0	3	1	0.264
Ependymoma	1	0	0	0	1	0.541
Malignant tumors	9	3	2	0	14	˂0.001
Prostate adenocarcinoma metastasis	6	1	2	0	9	0.049
Breast adenocarcinoma metastasis	2	1	0	0	3	0.070
Seminoma metastasis	1	0	0	0	1	0.596
Lung cancer metastasis	0	1	0	0	1	0.014

Evolution of the SCIM score: The evolution of the SCIM score mirrors that of the ASIA score. The majority of patients with malignant tumors, particularly those with prostate adenocarcinoma metastases (p = 0.010), remained dependent with a SCIM score ≤ 40 (p < 0.001). In contrast, patients with benign tumors, specifically meningiomas (p = 0.002), improved favorably and became independent in daily activities with a SCIM score ≥ 40 (p < 0.001).

## Discussion

General characteristics

In this study, the majority of patients were male (55.9%) and under 50 years of age (52.9%). The clinical presentation was dominated by pain corresponding to the location of the lesion (64.7%), neurological deficit in the form of incomplete paraplegia (76.6%), ASIA grade B (76.6%), and SCIM scores below 40% (79.4%), as well as vesico-sphincter disorders (41.2%). The average preoperative delay was 124.18 ± 73.34 days, approximately four months. Many spinal tumors were located in the thoracic segment (55.9%), with a predominance of intradural and extramedullary lesions. The most common procedures were laminectomy + total tumor excision (55.9%) and simple decompression laminectomy (23.5%). The average postoperative hospitalization duration was 26.32 ± 17.62 days. The majority of patients (67.6%) improved their entry ASIA score, with most improving by two grades (13/23: 56.3%). All ASIA grade C patients (100%) progressed by two grades. Patients with an initial SCIM score above 40 improved more than those with lower scores (71.8% vs. 66.6%, respectively).

In their study of 531 cases, Bhat et al. [15] documented a borderline male predominance (297/531 = 55.93%) with a sex ratio of 1.26. Iravanpour et al. [16] and Zabsonre et al. [17] confirmed male predominance in their reports with 59.6% and 73.6%, respectively. Other studies have reported a higher frequency of female subjects [18-20]. We believe that the discrepancy with our results could be explained by regional differences. Preston-Martin [20] found a female predominance in studies conducted in America. In contrast, studies conducted in Asia showed a higher proportion of male subjects. The closer one is to Asia, the more marked the male predominance.

Our results differ from some previous studies regarding patient age. In most studies, patients over 50 years of age predominate, unlike in our series [16,17]. This may be due to the sample size and the large number of benign tumors in our study. Additionally, in Africa, in general, and in the Democratic Republic of the Congo, in particular, the majority of the population is young. Zabsonre et al. [17] found an average age of 36 ± 16.67 years.

Clinically, our results are consistent with medical literature. Radiculomedullary compressions often present with acute pain corresponding to the lesion location, severe neurological deficits (motor or sensory), and vesico-sphincter disorders (urinary or fecal incontinence, urinary retention, or constipation) [21,22]. Randhawa et al. [7] documented 38.5% pain, followed by limb weakness (31.5%), paresthesia/numbness (22.3%), and sphincter disorders (7.7%). Fathy et al. [21] reported 14/16 cases of pain (87.5%), followed by 9/16 cases of sensory disturbances (56.2%) and 6/16 cases of motor deficits (37.5%). Iravanpour et al. [16] found 76.1% of cases with pain, followed by 28.4% with motor disturbances, 10% with sensory disturbances, and 9% with sphincter disorders. Zabsonre et al. [17] reported opposite findings, with functional impairment of one or more limbs in 89.4% (17/19) of cases, followed by back pain in two cases. We believe that pain is the initial sign of radiculomedullary tumor compression, and sensory-motor and sphincter disturbances are more progressive as the nerve impulse transmission is interrupted [6]. Since our patients tend to consult the hospital late, pain may be overshadowed by the deficit signs.

The data from this study align with medical literature regarding the thoracic location of spinal tumors as the most common [17-21,23].

The preoperative delay is often long. Tumor pathologies create a picture of slow medullary compression that gradually develops over days, weeks, and months. This situation becomes critical in less developed countries with limited diagnostic resources, such as MRI, which is not always available. This is the case in the Democratic Republic of Congo, where MRI exams are not always accessible. Arnautovic et al. [23] and Zabsonre et al. [17] reported average preoperative delays of 12 months and 78 days, respectively.

Our results differ from the majority of earlier studies that noted a predominance of extradural tumors, mainly consisting of metastases in 55% to 60% of cases [10]. The high frequency of intradural and extramedullary tumors found in this study was also reported by Zabsonre et al. [17] (9/19 cases, 63.6%) and Iravanpour et al. [16] (64/109 cases, 58.71%) for intradural and extramedullary tumors. We believe this discrepancy could be explained by the sample size. The predominance of benign tumors is mainly due to the fact that most patients with metastases die before reaching the hospital, as they undergo a prolonged diagnostic journey.

Patients with radiculomedullary tumor compressions generally have a favorable outcome, especially those with low-grade benign tumors, those who consult the hospital early, and those who present with moderate neurological deficits [10,22], as evidenced by our results. Fathy et al. [21] recorded improvements in 14/16 patients (87.5%). Dahal et al. [18] found 54.05% clinical improvement within a week.

Histopathological data

Benign tumors were more frequent than malignant ones (58.8% vs. 41.2%). They predominated in female subjects (p = 0.002) and those under 50 years old (p = 0.017). The most common benign lesions were meningiomas (47.1%). Malignant tumors were more frequently observed in male subjects (p = 0.002) over 50 years old (p = 0.017). All malignant tumors were metastases (14/14, 100%). Adenocarcinoma metastases were the most frequent (9/14, 64.2%), followed by those from breast adenocarcinoma (3/14, 21.4%). One case of prostate cancer metastasis was located at two different sites: thoracic and lumbar. We noted one case of recurrence. It was a high-grade ependymoma (grade III), the only primary intramedullary tumor.

Patients with benign tumors, particularly meningiomas, had significantly better neurological outcomes, gaining two to three grades more compared to their admission status (p < 0.001), with many gaining some autonomy (p < 0.001). In contrast, patients with metastases, such as prostate adenocarcinoma metastases (7/11, 63.7%), had unfavorable outcomes (p = 0.010) and remained dependent (p < 0.001). No case of metastasis improved by three grades or more.

Our results align with some previous studies that reported the predominance of benign tumors over malignant ones in the spine. These benign tumors were generally intradural and extramedullary [15,16]. Bhat et al. [15] documented 67.4% benign tumors versus 32.6% malignant ones. Among these tumors, 68.7% were intradural-extramedullary, 14.8% were intradural-intramedullary lesions, and 16.4% were both extradural and intradural tumors, mostly malignant. Iravanpour et al. [16] found 58.7% intradural tumors, including 36.6% extramedullary lesions and 22% intramedullary tumors. They also reported 41.3% extradural tumors. In contrast, most studies attest to the high frequency of malignant tumors, mostly extradural, represented by metastases [2,3,23,24].

The results of this study corroborate previous studies regarding the predominance of meningiomas in intradural-extramedullary lesions frequently found in female subjects [17]. However, most medical data place schwannomas as the leading cause of intradural-intramedullary tumors [18,19,22,23]. The high frequency of meningiomas in females is thought to be related to estrogen and progesterone, as their receptors are abundantly found in meningiomas. However, more randomized studies are needed to confirm this hypothesis [25].

The high rate of prostate adenocarcinoma metastases found in this study is close to that reported in previous studies [26-30]. However, this result contradicts general medical literature [31]. According to frequency order, breast cancer metastases (21%) are much more common in the spine, followed by those from the lung (14%), prostate (8%), and kidney (5%) [32]. This discrepancy could be explained by the small sample size in our study. Furthermore, the study was not conducted in the general population; only symptomatic patients who came to consult at the university teaching hospital of Kinshasa were included.

The location of prostate cancer metastases in two segments of the spine found in our study aligns with the literature, which highlights the predominance of single-site spinal metastases over multiple sites. The presence of metastases on multiple vertebrae from different spinal segments, and even outside the spine, is frequent in small-cell prostate carcinoma, a rare and aggressive histological entity (10%) [33]. The preference of prostate cancer for spinal metastases is thought to be facilitated by the Batson venous plexus, a valveless vein system located in the epidural space between the spine and dura mater [34].

The single case of ependymoma recurrence reported illustrates its existence in our setting and its high recurrence potential among intramedullary neoplasms [21].

Regarding postoperative outcomes, our results align with medical literature. Patients who underwent surgery for tumor-induced radiculomedullary compression typically have excellent outcomes with significant improvement in neurological function. The risk of recurrence is very low, except in cases of subtotal resection [35]. Complete resection of spinal meningiomas with a very short preoperative duration, performed in younger patients, generally yields good neurological results [36,37], unless the meningiomas are of high grade [38].

In contrast, surgery alone for malignant spinal tumors, mostly metastases, leads to less satisfactory neurological outcomes with a very high risk of recurrence. Early combination of surgery with radiotherapy is needed for a good neurological result and to reduce the recurrence rate [39]. Resection of vertebral metastases from prostate adenocarcinoma often leads, as shown in our results, to unfavorable neurological outcomes in regions where the current therapeutic protocol is not applied [26]. Our patients underwent surgery late after the disease onset, and six of them (6/14, 42.8%) did not receive radiotherapy. Therefore, the outcome after one year was less satisfactory. Adeolu et al. [26] reported 49% neurological improvement. Generally, if the therapeutic protocol for metastatic radiculomedullary compression (a combination of chemotherapy, surgery, and early radiotherapy) is followed, short- and medium-term therapeutic outcomes are often favorable from a neurological perspective [17].

Limitations and strengths of the study

The small sample size of the study and its monocentric nature, conducted solely at the university teaching hospital of Kinshasa, limit the generalization of the results. Other limitations are selection bias (patients with advanced metastases may have been excluded due to poor surgical candidacy) and the lack of long-term follow-up (especially for patients who underwent subtotal resection, who are likely to develop recurrences). Nevertheless, this study provides the first insight into spinal tumors in the Democratic Republic of the Congo.

## Conclusions

This study shows that benign tumors are more frequent than malignant tumors among patients with tumor-related radiculomyelopathy who underwent surgery at our healthcare institution. They predominantly affect females under the age of 50 years. Meningiomas are the most common tumors found among intradural and extradural lesions. Metastases are the most frequent malignant tumors, with prostate adenocarcinoma being the most common histopathological type. Neurological outcomes depend on whether the tumor is malignant or benign.
